# Invasive and Echocardiographic Mean Transvalvular Pressure Gradients of Different Transcatheter Aortic Valve Prostheses

**DOI:** 10.3390/jcm14165875

**Published:** 2025-08-20

**Authors:** Georges El-Hachem, Marcus-André Deutsch, Sebastian Rojas, Lech Paluszkiewicz, Mohammad Sharaf, Tomasz Gilis-Januszewski, Tanja Katharina Rudolph, Smita Scholtz, Kai Peter Friedrichs, René Schramm, Volker Rudolph, Jan Fritz Gummert, Dragan Opacic, Sabine Bleiziffer

**Affiliations:** 1 Clinic for Cardiac Surgery, Heart and Diabetes Center NRW, 32545 Bad Oeynhausen, Germany; mdeutsch@hdz-nrw.de (M.-A.D.); srojashernandez@hdz-nrw.de (S.R.); lpaluszkiewicz@hdz-nrw.de (L.P.); msharaf@hdz-nrw.de (M.S.); tgilis-januszewski@hdz-nrw.de (T.G.-J.); rschramm@hdz-nrw.de (R.S.); jgummert@hdz-nrw.de (J.F.G.); dopacic@hdz-nrw.de (D.O.); sbleiziffer@hdz-nrw.de (S.B.); 2 Clinic for General and Interventional Cardiology, Heart and Diabetes Center NRW, 32545 Bad Oeynhausen, Germany; trudolph@hdz-nrw.de (T.K.R.); sscholtz@hdz-nrw.de (S.S.); kpfriedrichs@hdz-nrw.de (K.P.F.); vrudolph@hdz-nrw.de (V.R.)

**Keywords:** transcatheter aortic valve replacement, balloon-expandable valve, self-expandable valve, invasive catheterization mean pressure gradient, echocardiographic mean pressure gradient, differential delta pressure

## Abstract

**Background/Objectives:** This study aimed to assess the effectiveness and clinical relevance of intraprocedural invasive measurements—specifically intraprocedural mean pressure gradients (IC MPGs) and diastolic delta (DD)—in comparison with echocardiography for evaluating transcatheter heart valve (THV) performance across different prosthesis types. Particular attention was paid to comparing outcomes between balloon-expandable (BE) and self-expandable (SE) valves, with further stratification by aortic annulus size. **Methods:** A retrospective analysis was performed on 926 patients who underwent transcatheter aortic valve replacements (TAVRs) between 2012 and 2021. Patients were categorized into BE (n = 301) and SE (n = 625) valve groups. Intraprocedural MPG was measured immediately before and after valve deployment. Postprocedural echocardiographic MPG (EC MPG) and the degree of aortic regurgitation were assessed within five days after implantation. Aortic annuli were classified as small (≤23 mm) or large (≥24 mm). **Results:** After implantation, EC MPG was consistently higher than IC MPG, with only a weak correlation observed between the two modalities. SE valves were generally associated with lower EC MPG than BE valves. DD was higher in the BE group; however, no significant correlation was found between DD and echocardiographically assessed aortic regurgitation. **Conclusions:** Intraprocedural invasive measurements offer a reliable and immediate assessment of prosthesis function during TAVR but tend to underestimate gradients compared to echocardiography. Newer SE valves show performance comparable to BE valves, particularly in small annuli, supporting their use in challenging anatomies. DD appears to lack a diagnostic value for postprocedural aortic regurgitation.

## 1. Introduction

As the low-intermediate-risk category gains approval for transcatheter aortic valve replacement (TAVR), the assessment of long-term valve function becomes pivotal in selecting the appropriate valve type. Recent findings from trials like Evolut Low-Risk and PARTNER 3 underscore the increasing relevance of valve durability and functionality, particularly when considering implantation in younger individuals [1,2]. In steering TAVR utilization towards these patients, attaining low post-interventional mean pressure gradients (MPGs) and minimizing paravalvular leakages (PVLs) stand as paramount objectives, along with the avoidance of required pacemaker implantations. The foundational step necessitates delving into real-world data to evaluate the outcomes associated with different prostheses, facilitating the tailored identification of the most suitable prosthesis for each individual patient. Among transcatheter heart valve (THV) prostheses, the commonly employed types are the balloon-expandable (BE) and self-expandable (SE) valves. The SE valves exhibit associations with lower gradients, notably in smaller anatomies, while BE valves tend to showcase lower rates of paravalvular leakages [3,4].

The optimal approach for quantifying PVLs and transvalvular MPGs continues to be a subject of debate. While echocardiography stands as the most practical non-invasive method for assessing PVL and transvalvular MPG [5], its calculations rely on aortic blood flow velocity, susceptible to influences from factors like blood viscosity or concurrent left ventricular outflow tract (LVOT) stenosis and left ventricular function [6]. On the other hand, cardiac catheterization directly measures MPG, reducing susceptibility to errors. Furthermore, utilizing echocardiography to quantify PVL post-TAVR poses challenges due to intricate regurgitation patterns observed in these patients [7]. Recent suggestions propose that a low differential delta (DD), depicting the difference between end-diastolic aortic pressure and left ventricle end-diastolic pressure (LVEDP), could better indicate significant PVLs [8]. Therefore, determining the precise discrepancy between echocardiography-derived MPGs and invasively measured MPGs as well the quantification of PVLs, are crucial to ascertain their clinical significance.

Our study aims to analyze intraprocedural invasive measurements of transvalvular mean pressure gradients across various THV prostheses immediately after implantation. Specifically, our objectives are to (1) assess the predictive value of invasive measurements in relation to echo-derived gradients, (2) provide tables depicting expected average values for different valve types, and (3) stratify these values based on annulus size. Moreover, in the pursuit of identifying patients with noteworthy PVL, (4) we employ the differential delta (DD) of distinct THV prostheses immediately post-implantation and compare it to subsequent echocardiographic PVL findings.

## 2. Materials and Methods

This research project was reviewed in advance by the Ethics Committee of the Ruhr University Bochum Bad Oeynhausen (Az:2022-898) and a positive vote was obtained. All methods were carried out in accordance with relevant guidelines and regulations. Additionally, all experimental protocols were approved by the Ethics Committee of the Ruhr University Bochum Bad Oeynhausen. Informed consent was obtained from all subjects and/or their legal guardian(s).

We conducted a retrospective evaluation of 3909 TAVR patients treated at our center between January 2012 and December 2021. Patients were excluded if they underwent a valve-in-valve procedure, if invasive measurement data were missing, or if they received valves other than those listed in this analysis. Of these, 926 patients had available invasive hemodynamic data and met our inclusion criteria (Supplementary Figure S1). The different prostheses used were balloon-expandable Sapien 3 and Sapien 3 Ultra (Edwards Lifesciences, Irvine, CA, USA) and self-expandable valves Evolut R, Evolut Pro (Medtronic Inc., Dublin, Ireland), and Acurate, Acurate Neo, and Acurate Neo 2 (Boston Scientific—Symetis, Marlborough, MA, USA). Patients treated with Symetis Acurate and Acurate Neo were combined in one group (Acurate Neo).

### 2.1. Invasive Gradient

Immediate transvalvular mean gradients were obtained by invasive hemodynamic assessment (Sensis Vibe, Siemens, Erlangen, Germany). Invasive assessments of left ventricular (LV) and aortic pressures as well as the mean transvalvular gradient were performed simultaneously with two single-lumen pigtail catheters immediately before and after valve deployment. The LV pressure was obtained as apically as possible using a pigtail catheter inserted into the LV while the aortic pressure was obtained in the ascending aorta from another pigtail catheter that was placed via the right radial artery or a contralateral femoral artery. Both catheters were adequately flushed and zeroed, and waveforms were checked for attenuation before recording. The DD was determined as a difference between end-diastolic aortic blood pressure and left ventricle end-diastolic pressure (LVEDP).

### 2.2. Echocardiography

Postoperative transthoracic echocardiographic evaluation of the mean pressure gradient (EC MPG) was performed within 3 to 5 days after the intervention, using a commercially available Philips EPIQ diagnostic ultrasound system (Philips Medical Systems, Andover, MA, USA), in accordance with current protocols and the European guidelines by Baumgartner et al., 2017 [9].

Echocardiographic grading of PVL was based on an integrative multi-parametric approach that mainly included a visual assessment of the number of PVL jets, jet width at the origin, and the circumferential extent of PVL [10]. The degree of PVL was classified into none/trace, mild, moderate, or severe.

### 2.3. Aortic Annuli

Aortic annulus measurements were obtained from TAVR protocol computed tomography (3Mensio Medical Imaging, Utrecht, The Netherlands). To date, no clear consensus has been established regarding the cutoff value for defining a small annulus. An aortic annulus ≤ 23 mm, measured either preoperatively or intraoperatively by direct sizing, has been proposed as the definition of a small annulus [11]. We utilized this definition in our study, categorizing an aortic annulus ≤ 23 mm as “small annulus” and an annulus ≥ 24 mm as “large annulus” based on computed tomography measurements.

### 2.4. Statistics

The continuous variables are presented as median and interquartile range, while categorical variables are presented as percentages. The Mann–Whitney U test and Wilcoxon test were used to compare continuous variables. Categorical variables were compared using the chi-squared test and Fischer’s exact test. The correlation between invasively and non-invasively measured gradient over the aortic valve was evaluated with Spearman’s correlation, Lin’s Concordance coefficient, and the Bland and Altman diagram.

Cox regression employing a conditional backward stepwise removal method was used to assess the effects of preoperative parameters, pacemaker implantation rate, and postoperative aortic regurgitation greater than mild, as well as a difference in MPG of more than 10 mmHg between echocardiographic and invasive measurements, on long-term survival. Kaplan–Meier curves were used to depict survival over time.

A *p*-value ≤ 0.05 was considered significant. Statistical analysis and data presentation were performed with SPSS Software (IBM SPSS Statistics for Windows, Version 29.0, IBM Corp. Armonk, NY, USA) and GraphPad Software (GraphPad Prism version 10 for Windows, San Diego, CA, USA).

## 3. Results

Of the 3909 patients who had undergone TAVR, 957 had paired echocardiographic and invasive hemodynamic measurements. After applying exclusion criteria, 926 patients were included in the analysis. The median age was 81 years; 78–85 years, and the gender distribution was similar (445 (48.1%) were males and 481 (51.9%) were females). Preoperative echocardiography-derived parameters showed a median effective orifice area of 0.7; 0.6–0.9, while LVEF was 55; 50–55%. Transfemoral access was used in 98.2% of patients (Table 1).

The median invasive catheterization gradient (IC MPG) before intervention was 43; 34–53 mmHg, while LVEDP was 15; 11–21 mmHg. After the procedure, the IC MPG was significantly reduced (5; 3–7 mmHg), but LVEDP was slightly but still significantly increased (18; 13–23 mmHg) (Table 2).

The postoperative pacemaker implantation rate was 12.2%, ranging from 3.4% for the Symetis Accurate Neo 2 to 18.5% for the Medtronic Evolut Pro.

Table 3 depicts both IC and EC MPG for each valve type and size implanted. The most commonly used SE valve was Evolut R, implanted in 304 patients (32.8%), while the most frequently used BE valve was Sapien 3, which was received by 244 patients (26.3%).

**Figure 1:**
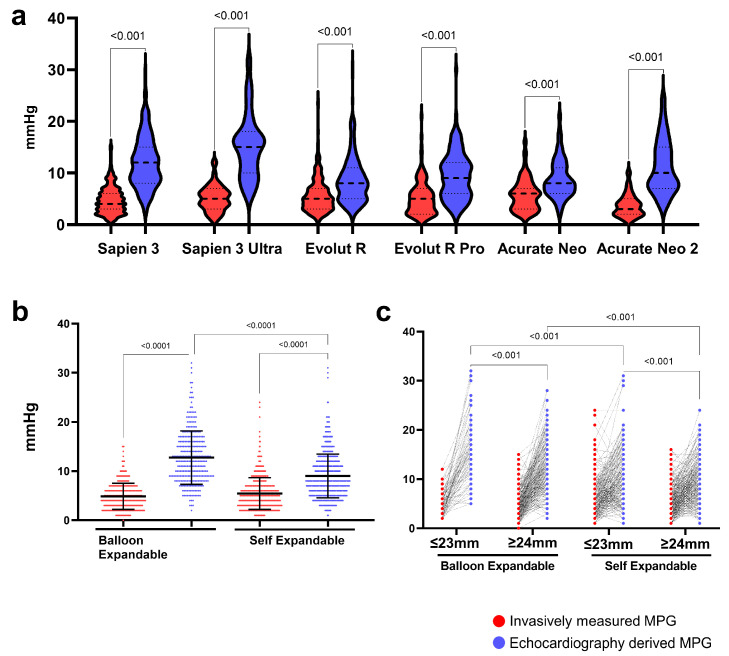
**Invasively measured (IC) and echocardiography (EC)-derived mean pressure gradients for the different valve types.** (**a**) IC and EC measures of the MPG of the different heart valves, (**b**) comparisons of IC and EC-derived MPGs of BE and SE valves, and (**c**) comparison of invasively measured and echocardiography-derived mean pressure gradients between balloon-expandable and self-expandable valves in patients with small (≤23 mm) and large annuli (≥24 mm).

**Figure 2:**
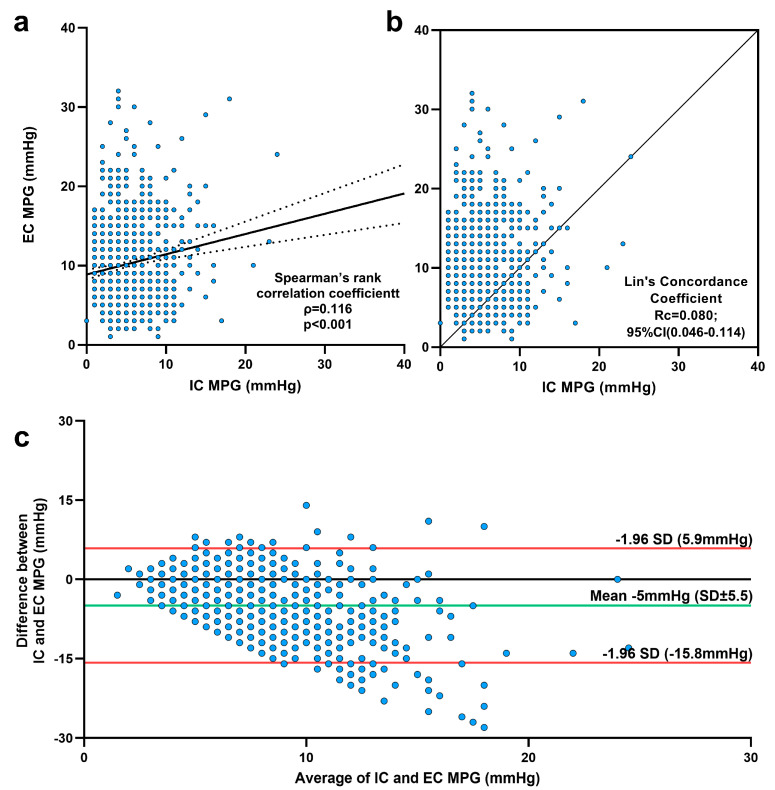
**Comparisons of intraprocedural (IC) and echocardiographic (EC) mean pressure gradients (MPGs)**. (**a**) Spearman’s rank correlation analysis demonstrated a statistically significant correlation between postinterventional IC and EC MPG; the association was very weak. (**b**) Lin’s concordance coefficient between IC MPG and EC MPG. (**c**) Bland and Altman diagram.

The EC MPG had generally higher values than IC MPG, which was observed across all used valves (Figure 1a). After the grouping to balloon-expandable (Sapien 3 and Sapien 3 Ultra) and self-expandable valve types (Evolut R, Evolut Pro, Acurate Neo, and Acurate Neo 2), the EC MPG was significantly higher than the IC MPG both in BE (12; 9–16 mmHg vs. 5; 3–6 mmHg, *p* < 0.001) and SE (8; 6–11 mmHg vs. 5; 3–7 mmHg, *p* < 0.001) valves (Figure 2b). Furthermore, we investigated the effect of the annulus size on the postoperative gradients. Patients with a large annulus (≥24 mm) had a lower EC MPG in balloon-expandable and self-expandable valves, while the IC MPG was similar. Importantly, self-expandable valves had lower EC MPGs both in patients with small (≤23 mm) and large annuli (≥24 mm) (Figure 1c). The EC MPG was significantly higher than the IC MPG both in patients with small (≤23 mm) and large (≥24 mm) annuli. The IC MPG within the same valve group was similar between patients with small and large annuli, but the EC MPG was significantly higher in small-annulus patients in all valves except Evolut Pro and Acurate Neo 2 (Supplementary Figure S2).

Although the EC MPG values were statistically higher across all valve groups, the correlation between the EC MPG and IC MPG, as assessed by Spearman’s rank correlation, was significant but still very weak. Lin’s concordance correlation indicated a poor agreement between the two measurements (Figure 2b). However, the Bland–Altman analysis (Figure 2c) showed that EC MPG values were indeed approximately 5 mmHg higher than IC MPG, but nearly all differences between EC and IC MPG fell within the 95% limits of agreement. Only 34 patients (3.7%) had EC MPG values exceeding the agreement limit (>15 mmHg), whereas 137 patients (14.8%) had an EC MPG at least 10 mmHg higher than the IC MPG. The Cox regression model deemed EC MPG values greater than both 10 mmHg and 15 mmHg as irrelevant for long-term survival (Supplementary Table S1).

The median survival of all patients was 63 months (95% CI: 59–73 months), while 440 out of 900 patients (47.5%) had complete follow-up data at 5 years.

**Figure 3:**
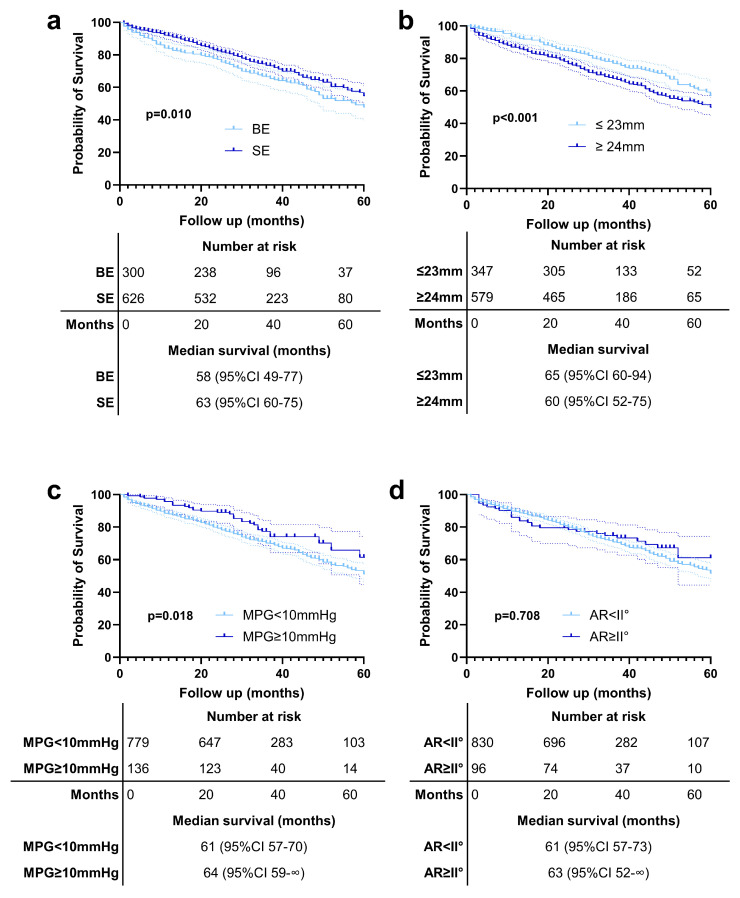
Kaplan–Meier survival curves comparing outcomes between (**a**) patients with balloon-expandable (BE) and self-expanding (SE) valves, (**b**) patients with small versus large annuli, (**c**) patients with an EC MPG and IC MPG difference > 10 mmHg, and (**d**) those with aortic regurgitation (AR) ≥ grade II°.

In Figure 3, we present Kaplan–Meier survival curves comparing outcomes between patients with BE and SE valves (Figure 3a), patients with small versus large annuli (Figure 3b), patients stratified by differences between IC MPG and EC MPG values greater than 10 mmHg (Figure 3c), and those with aortic regurgitation ≥ grade II° (Figure 3d). It is important to note that these survival curves are unadjusted and should therefore be interpreted with caution, as they do not account for potential confounding variables.

Our analysis showed that patients receiving SE valves had longer survival compared to those with BE valves (Figure 3a). Similarly, patients with small annuli demonstrated better survival outcomes than those with large annuli (Figure 3b). Interestingly, we observed that patients with a difference in MPG > 10 mmHg between EC and IC measurements had significantly better survival compared to those without such a difference (Figure 3c). Further subgroup analysis revealed that this effect was primarily driven by patients who received BE valves; however, the significant survival benefit was observed only in patients with a large annulus (Supplementary Figure S3).

At discharge, the majority of patients had either none (476; 51.4%) or mild (354; 38.2%) outcomes. Moderate AR was observed in 95 (10.3%), while only one patient had severe AR at discharge. We categorized AR as none to mild versus greater than or equal to moderate, as moderate AR is considered to have relevant long-term consequences. The PVL/AVR data for each valve type are presented in Table 4.

**Figure 4:**
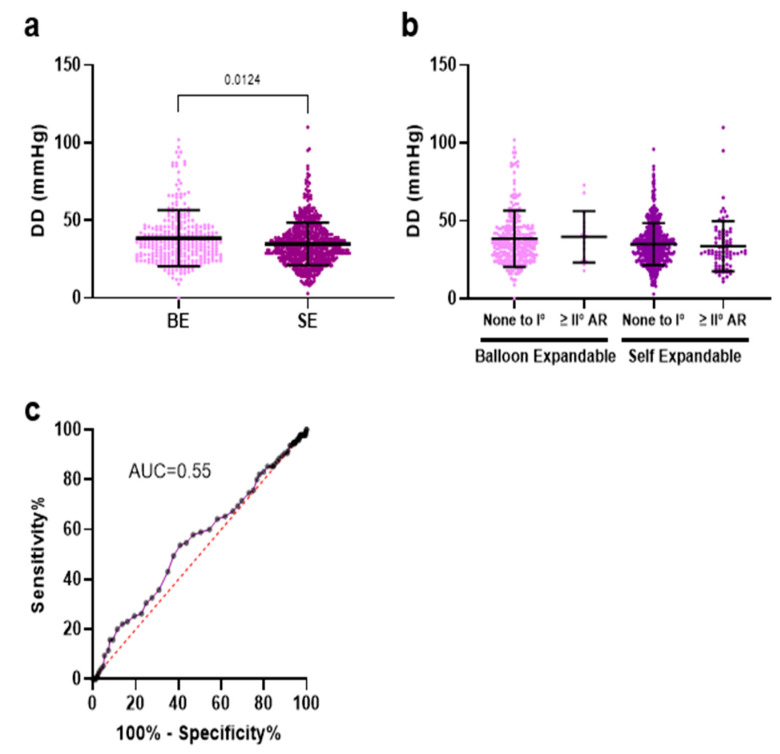
(**a**) Differential delta (DD = diastolic AoP—LVEDP) was significantly higher in the BE group compared to SE valves. (**b**) AR as none to mild versus greater than or equal to moderate, as moderate AR. (**c**) DD was not predictive for relevant echocardiographic postinterventional AR.

Patients receiving BE valves exhibited a significantly lower incidence of PVL of any severity when compared to those treated with SE valves. BE valves had significantly higher DD values than SE valves (35; 27–45 vs. 34; 26–41 mmHg, *p* < 0.01) (Figure 4a). We categorized AR as none to mild versus greater than or equal to moderate, as moderate AR is considered to have relevant long-term consequences. However, there was no significant difference in DD between patients with mild and greater-than-mild AR, regardless of the valve types used (Figure 4b). Furthermore, the predictive value of postprocedural DD for relevant (greater than mild) AR was insignificant (Figure 4c). Annulus size was irrelevant for the DD within the same prosthesis types (Supplementary Figure S4).

## 4. Discussion

Our study demonstrated that EC MPGs were slightly but still significantly higher than IC MPGs independently of the valve type used and annulus size. Although EC and IC MPG showed only a weak correlation post-implantation, the differences between EC MPG and IC MPG were within the limits of agreement and did not affect long-term outcomes; thus, they can be used interchangeably. Notably, among the valve types studied, SE valves exhibited significantly lower EC MPGs in patients with both small and large annuli compared to BE valves. Finally, our findings do not support the predictive utility of the DD in anticipating postoperative echocardiographic paravalvular regurgitation.

The phenomenon of the overestimation of transvalvular mean gradients from EC compared to IC is a well-established occurrence in native aortic stenosis [8]. This discrepancy is an inherent limitation of Bernoulli’s equation when applied to normally functioning prostheses, extending beyond the effects of pressure restoration [12]. The overestimation of the EC compared to IC MPG was consistent in all prosthetic valve types used in our study.

Several studies have shown a correlation between echocardiography and invasive mean gradients in native aortic valve stenosis [9], a correlation that diminishes following treatment of the stenotic valve [13,14]. Our dataset corroborates this trend, depicting a statistically significant but weak correlation between EC and IC MPG after stenosis treatment. However, Bland–Altman analysis showed that the differences between the two methods were small, suggesting that the weak correlation is rather due to inherent variability within each measurement technique than a systematic bias. Therefore, we concluded that although echocardiography tends to report slightly higher MPG values, the difference between EC and IC lies within the expected internal variability of both modalities, indicating that the two methods are comparable and can be used interchangeably in clinical practice. Notably, the slight disparity between EC MPG and IC MPG remains consistent across valve annulus sizes, as confirmed by other studies [15].

The critical question remains if the gradients, whether measured invasively or via echocardiography, are associated with patient outcomes. According to a large retrospective multicenter study by Khalili et al., the impact of transaortic gradients on mortality after TAVR is very complex and depends much on left ventricular function, showing opposite results between echocardiographic and invasive measurements in low-gradient patients [16]. Therefore, more comprehensive studies are needed to determine the relationship between gradient measurements and clinical outcomes, to improve predictive value for patient prognosis post-TAVR.

For small (≤23 mm) and large annuli (≥24 mm), there was a significant difference in EC MPGs between BE valves and SE valves with lower gradients in the SE valve group. This observation was also made in other studies such as the randomized CHOICE Trial [17] and others [18]. The supra-annular valve design inherent to the SE prostheses used in our study may account for these lower gradients. Remarkably, the latest generation SE prostheses exhibited consistently low MPGs. This trend was confirmed even in patients with small annuli in the recently published results of the randomized SMART trial [4]. In this trial, patients receiving SE valves had lower EC MPGs compared to BE prostheses, although this difference was not observed in IC MPGs. Furthermore, the SE valves were non-inferior to the BE valves in terms of clinical outcomes. However, it is important to note that clinical outcomes were assessed based on EC MPG and not IC MPG, which could have affected the results. Additionally, the relatively short follow-up period in that trial may have been insufficient to detect differences in clinical outcomes between the valve types.

In line with these findings, a recent real-world study evaluating the performance of a new-generation SE valve (Portico with FlexNav delivery system) reported favorable one-year outcomes, including low post-TAVR gradients, preserved valve area, and significant clinical improvement [19].

Our findings, indicating lower gradients with SE valves in small annuli, coupled with the provided charts of predicted average gradients, may serve as valuable guidance in individual valve selection for TAVR. This decision-making process should consider anatomical suitability, risk of patient-prosthesis mismatch, patient lifestyle, and life expectancy.

The association between PVL and patient mortality is well-documented, with patients exhibiting moderate or severe PVL having a threefold-higher mortality rate compared to those without evidence of PVLs [20,21]. Recently, even mild to moderate PVL has been linked to an increased risk of mortality at the 5-year mark post-TAVR [22]. Therefore, periprocedural evaluation of PVL holds paramount importance. Identifying relevant PVL during the TAVR procedure can guide subsequent interventions, such as post-dilatation, aimed at minimizing PVL and enhancing patient outcomes.

DD has emerged as a potential hemodynamic predictor of aortic regurgitation. Notably, the recent APPOSE trial highlighted DD as possessing the highest predictive value for relevant PVL (defined as CMR-RF > 20%) a month post-TAVR [8]. However, our data do not replicate these findings, indicating a lack of correlation between DD and echocardiography-assessed aortic regurgitation at discharge. It is important to note that we try to correlate the DD values with the echocardiographic findings, and transthoracic echocardiography faces limitations in accurately visualizing multiple eccentric jets, potentially leading to underestimations in EC measurements. Therefore, a multimodal approach involving echocardiography, angiography, and DD measurements appears most appropriate for a comprehensive evaluation of transcatheter valve regurgitation.

Since obtaining invasive mean gradients during long-term follow-up post-TAVR is impractical, echocardiography remains crucial for monitoring significant changes in mean gradients from immediately post-TAVR and for ongoing follow-up, especially for assessing aortic regurgitation. It is recognized that mean gradients measured at discharge are typically higher than those measured immediately post-TAVR. Larger ongoing studies are essential to evaluate the implications of elevated mean gradients.

### Limitations

The primary limitation of this analysis lies in its retrospective and non-randomized design, which inherently restricts the generalizability of the results. Invasive and echocardiographic measurements were performed under different hemodynamic conditions: during invasive gradient assessment, patients were sedated, whereas echocardiographic measurements were conducted several days post-intervention in already mobilized patients. Additionally, echocardiography was performed with patients in the left lateral recumbent position, whereas invasive measurements were obtained in the supine position. This difference in posture may have contributed to the observed discrepancies between echocardiographic and invasive values.

Furthermore, selection bias must be considered, as the data from only 926 out of approximately 4000 patients could be analyzed due to the reasons mentioned above, potentially limiting the representativeness of the findings.

An additional limitation that should be acknowledged is the comparison of post-TAVR echocardiographic parameters, such as valve area, across different valve types. Given the variability in valve design and implantation level (e.g., intra- vs. supra-annular), such comparisons may not fully reflect true hemodynamic differences.

## 5. Conclusions

This study highlights several key findings:

1. Post-TAVR, discharge EC MPGs are higher than IC MPGs measured immediately post-procedure, particularly in BEV compared to SEV, with smaller BEVs showing the highest gradients. This difference could be attributed to the significantly different settings in which both measurements are made.
2. The very small difference between invasively measured and echocardiographic-derived MPGs over the aortic valve resulted in, at first glance, a weak to non-existent correlation, irrespective of valve type. Importantly, the vast majority of measurements were within the limits of agreement, actually confirming the utility of both modalities.
3. Invasive mean gradients post-TAVR are similar across all valve sizes, but echocardiographically, smaller BEVs have higher gradients than larger BEVs. Both small and large SEVs show comparable echocardiographic gradients.
4. Both BEVs and SEVs demonstrate an excellent hemodynamic performance, with low mean gradients both immediately post-TAVR and at discharge.
5. The invasively measured differential delta did not predict echocardiographic paravalvular regurgitation, underscoring the need for a multimodal evaluation.

## Figures and Tables

**Table 1:** Patient characteristics.

Patient Characteristics	Results
Age (years)	81; 78–85
Men n, (%)	445 (48.1%)
BMI (kg/m^2^)	27.2; 24.3–30.4
NYHA Class	
I n, (%)	20 (2.2%)
II n, (%)	286 (30.9%)
III n, (%)	571 (61.7%)
IV n, (%)	49 (5.3%)
Diabetes mellitus n, (%)	323 (34.9%)
Dialysis n, (%)	18 (1.9%)
COPD n, (%)	164 (17.7%)
No CAD n, (%)	407 (44.0%)
CAD 1 Vessel n, (%)	195 (21.0%)
CAD 2 Vessel n, (%)	139 (15.0%)
CAD 3 Vessel n, (%)	185 (20.0%)
PAD n, (%)	99 (10,7%)
Atrial fibrillation n, (%)	271 (29.3%)
AVA (cm^2^)	0.7; 0.6–0.9
Valve annulus (mm)	24; 23–26
Annulus ≤ 23 mm n, (%)	347 (37.5%)
Annulus ≥ 24 mm n, (%)	579 (62.5%)
LVEF (%)	55; 50–55
Balloon dilatation before impl. n, (%)	318 (34.3%)
Balloon dilatation after impl. n, (%)	294 (31.7%)
TAVR TF n, (%)	909 (98.2%)

Abbreviations: BMI—body mass index; NYHA—New York Heart Association; COPD—chronic obstructive pulmonary disease; CAD—coronary artery disease; PAD—peripheral artery disease; AVA—aortic valve area; LVEF—left ventricular ejection fraction; TAVR—transcatheter aortic valve replacement.

**Table 2:** The different invasive parameters measured before and after valve implantation.

Parameter	Pre TAVR (mmHG) Median; IQR	Post TAVR (mmHg) Median; IQR	*p*
LVEDP	15; 11–21	18; 13–23	<0.01
Aortic pressure systolic	103; 89–120	124; 107–144	<0.01
Aortic pressure diastolic	50; 43–58	52; 45–62	<0.01
ICMPG	43; 34–53	5; 3–7	<0.01

Abbreviations: LVEDP: left ventricle end diastolic pressure; ICMPG: invasive catheterization mean pressure gradient.

**Table 3:** Invasive mean gradients and ultrasound-derived gradients of the different valves used.

Valves	No. Patients	Valve Size	No. Patients	IC MPG (mmHg) Median; IQR	EC MPG (mmHg) Median; IQR
Sapien 3	244 (26.3%)	23 mm	49	4; 2–6	15; 12–18.5
		26 mm	73	5; 3–7	11; 8–15
		29 mm	122	4.5; 3–6	11; 8–13
Sapien 3 Ultra	56 (6.0%)	23 mm	16	6; 4–7	16.5; 14.5–24
		26 mm	40	5; 3–7	14; 9–18
Evolut R	304 (32.8%)	23 mm	15	13; 10–16	17; 13–20
		26 mm	78	4.5; 3–7	7; 5–10
		29 mm	128	5; 3–7	8; 5–10
		34 mm	83	5; 4–7	8; 5–10
Evolut Pro	157 (17.0%)	26 mm	37	5; 3–7.5	10; 5.5–12.5
		29 mm	120	4; 2–7	9; 6–12
Acurate Neo	136 (14.7%)	S	42	6; 3–9	10; 8–13
		M	62	5; 3–7	8; 6–11
		L	32	5; 3–7	7; 6–8
Acurate Neo 2	29 (3.1%)	S	11	3; 2–4	11; 7–17
		M	12	4; 2–6	10.5; 8–14
		L	5	2.5; 2–5.5	8; 6.5–13.5

Abbreviations: IC MPG: invasive mean pressure gradient; EC: echocardiography-derived mean pressure gradient.

**Table 4:** Classification of postprocedural aortic regurgitation in balloon-expandable and self-expandable valves.

	None	Mild	Moderate	Severe
Balloon-expandable valves	193 (64.3%) †	92 (30.7%) †	15 (5.0%) †	0 (0.0%)
Sapien 3	153 (62.7%)	78 (32.0%)	13 (5.3%)	0 (0.0%)
Sapien 3 Ultra	40 (71.4%)	14 (25.0%)	2 (3.6%)	0 (0.0%)
Self-expandable valves	283 (45.2%) †	262 (41.9%) †	80 (12.8%) †	1 (0.2%)
Evolut R	154 (50.7%)	122 (40.1%)	28 (9.2%)	0 (0.0%)
Evolut Pro	66 (42.0%)	66 (42.0%)	25 (15.9%)	0 (0.0%)
Accurate neo	49 (36.0%)	59 (43.4%)	27 (19.9%)	1 (0.7%)
Acurate neo2	14 (48.3%)	15 (51.7%)	0 (0.0%)	0 (0.0%)
Total	476 (51.4%)	354 (38.2%)	95 (10.3%)	1 (0.1%)

†—*p* < 0.001 balloon-expandable vs. self-expandable.

## Data Availability

The data presented in this study are available on request from the corresponding authors due to data protection regulations and medical confidentiality.

## Supplementary Materials

The following supporting information can be downloaded at: https://www.mdpi.com/article/10.3390/jcm14165875/s1, Figure S1: Study protocol; Figure S2: Comparison of invasively measured and echocardiography-derived mean pressure gradients in the different valve types in patients with small (≤23 mm) and large (≥ 24 mm) annuli: (**a**) Sapien 3, (**b**) Sapien 3 Ultra, (**c**) Evolut R, (**d**) Evolut Pro, (**e**) Acurate Neo, and (**f**) Acurate Neo2; Figure S3: Kaplan–Meier survival curves comparing outcomes between patients with differences in EC MPGs and IC MPGs greater than 10 mmHg: (a) patients with a small annulus ≤ 23 mm receiving balloon-expandable valves, (b) patients with a small annulus ≥24 mm receiving balloon-expandable valves, (**a**) patients with a small annulus ≤ 23 mm receiving self-expandable valves, and (**b**) patients with a small annulus ≥ 24 mm receiving self-expandable valves; Figure S4: Comparison of invasively measured DD in the different valve types in patients with small (≤23 mm) and large (≥24 mm) annuli: (**a**) Sapien 3, (**b**) Sapien 3 Ultra, (**c**) Evolut R, (**d**) Evolut Pro, (**e**) Acurate Neo, and (**f**) Acurate Neo2; Table S1: Independent factors influencing long-term survival according to the Cox regression model.

## Abbreviations

The following abbreviations are used in this manuscript:

TAVR	Transcatheter aortic valve replacement
THV	Transcatheter heart valve
BE	Balloon-expandable
SE	Self-expandable
IC MPG	Invasive catheterization mean pressure gradient
EC MPG	Echocardiographic mean pressure gradient
DD	Differential delta
LV	Left ventricle
LVOT	Left ventricular outflow tract
LVEDP	Left ventricle end-diastolic pressure
EF	Ejection fraction
LVEF	Left ventricle ejection fraction
PVL	Paravalvular leakage
AR	Aortic regurgitation
