# Supplementary material for: Invasive and Echocardiographic Mean Transvalvular Pressure Gradients of Different Transcatheter Aortic Valve Prostheses
Source: J Clin Med. 2025 Aug 20;14(16):5875. doi: 10.3390/jcm14165875 (PMC12387880; doi:10.3390/jcm14165875)

Supplement

Supplementary tables

Supplementary Table S1. Independent factors influencing long term survival according to Cox regression model.

Variables	HR	95.0% CI	p
**Age**	1.075	1.053 - 1.098	<.001
**DM**	1.649	1.325 - 2.054	<.001
**Dialysis**	1.931	1.055 - 3.537	.033
**COPD**	1.620	1.268 - 2.070	<.001
**PAVK**	1.682	1.258 - 2.251	<.001
**AF**	1.503	1.198 - 1.884	<.001
**Large Annulus**	1.379	1.092 - 1.742	.007
**LVEF (%)**	.983	0.973 - 0.993	.001

Cox Regression employing a conditional backward stepwise removal method was used to assess the independent factors on long-term survival. Variables included in Cox regression model for long term survival: Sex, age, diabetes mellitus (DM), dialysis, chronic obstructive pulmonary disease (COPD), body mass index (BMI), coronary artery disease (CAD), peripheral artery occlusive disease (PAOD), atrial fibrillation (AF), large Annulus (≥24 mm), left ventricular ejection fraction (LVEF), postoperative aortic insufficiency greater than mild, postoperative pacemaker implantation, EC MPG larger than ICG MPG for at least 10 mmHg/15 mmHg.

Supplemental figures

Supplementary Figure S1. Study protocol

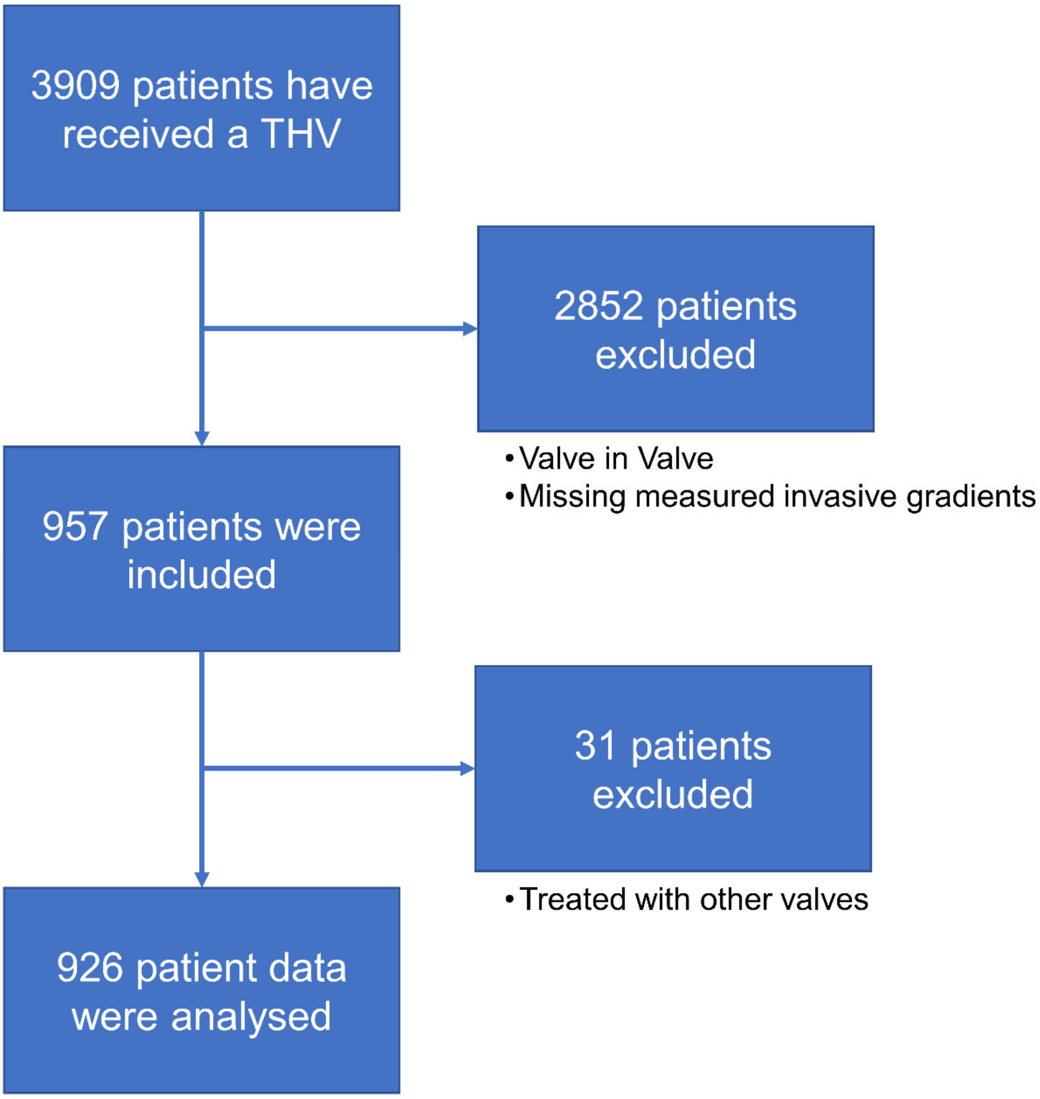

Supplementary Figure S2. Comparison of invasively measured and echocardiography derived mean pressure gradients in the different valve types in patients with small (≤23mm) and large (≥ 24mm) annuli. **(a)** Sapien 3, **(b)** Sapien 3 Ultra, **(c)** Evolut R, **(d)** Evolut Pro, **(e)** Acurate Neo, **(f)** Acurate Neo2

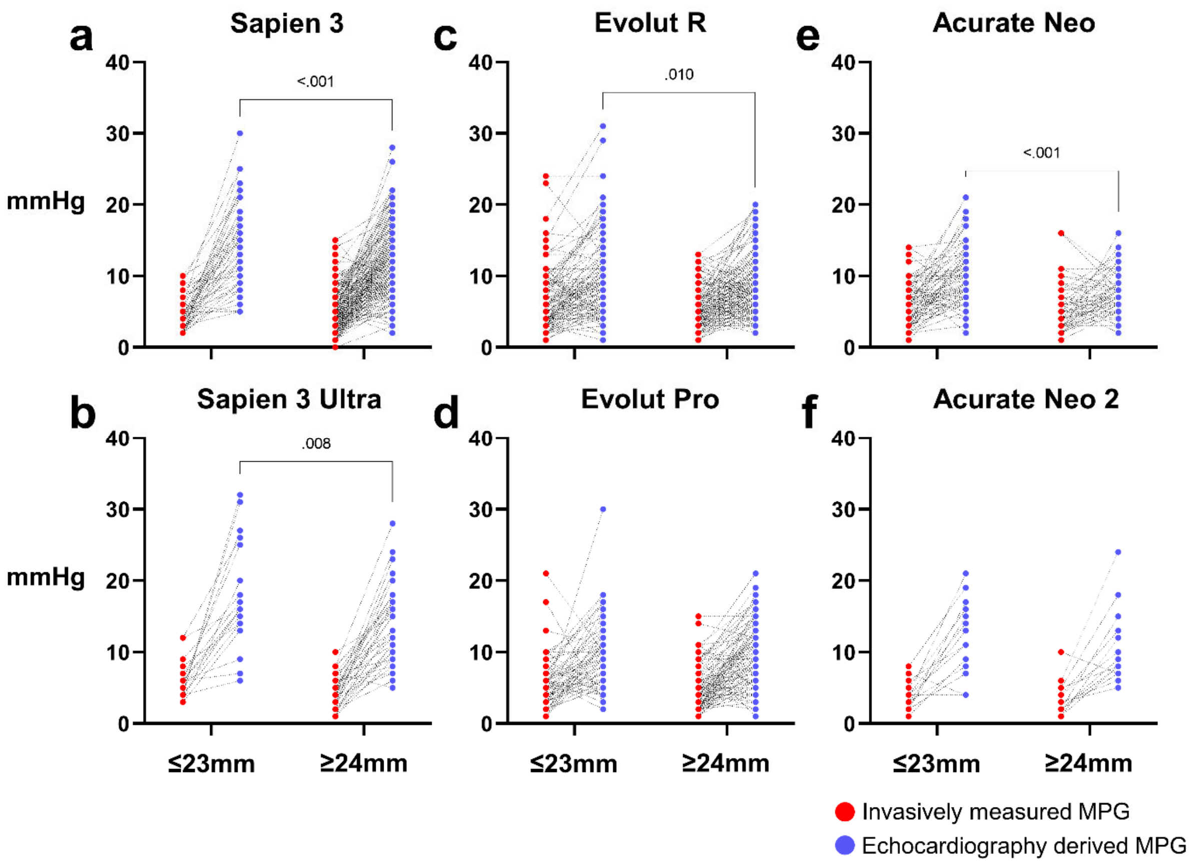

Supplemental Figure S3. Kaplan–Meier survival curves comparing outcomes between patients with difference in EC MPG and IC MPG greater than 10 mmHg (a) Patients with small annullus ≤23 mm recieveng balloon-expandable valves; (b) patients with small annullus ≥24 mm recieveng balloon-expandable valves; (a) patients with small annullus ≤23 mm recieveng self-expandable valves; (b) patients with small annullus ≥24 mm recieveng self-expandable valves.

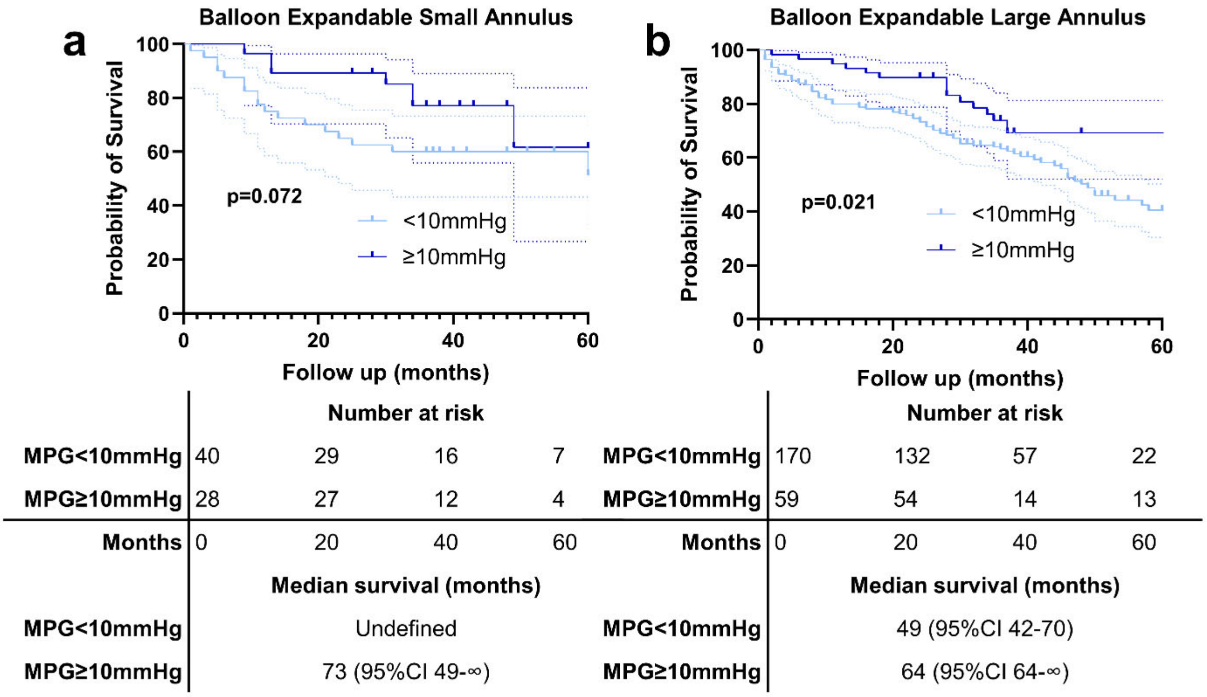

	Number at risk (a)					Number at risk (b)			
MPG<10mmHg	40	29	16	7	MPG<10mmHg	170	132	57	22
MPG≥10mmHg	28	27	12	4	MPG≥10mmHg	59	54	14	13
Months	0	20	40	60	Months	0	20	40	60

	Median survival (months) (a)		Median survival (months) (b)
MPG<10mmHg	Undefined	MPG<10mmHg	49 (95%CI 42-70)
MPG≥10mmHg	73 (95%CI 49-∞)	MPG≥10mmHg	64 (95%CI 64-∞)

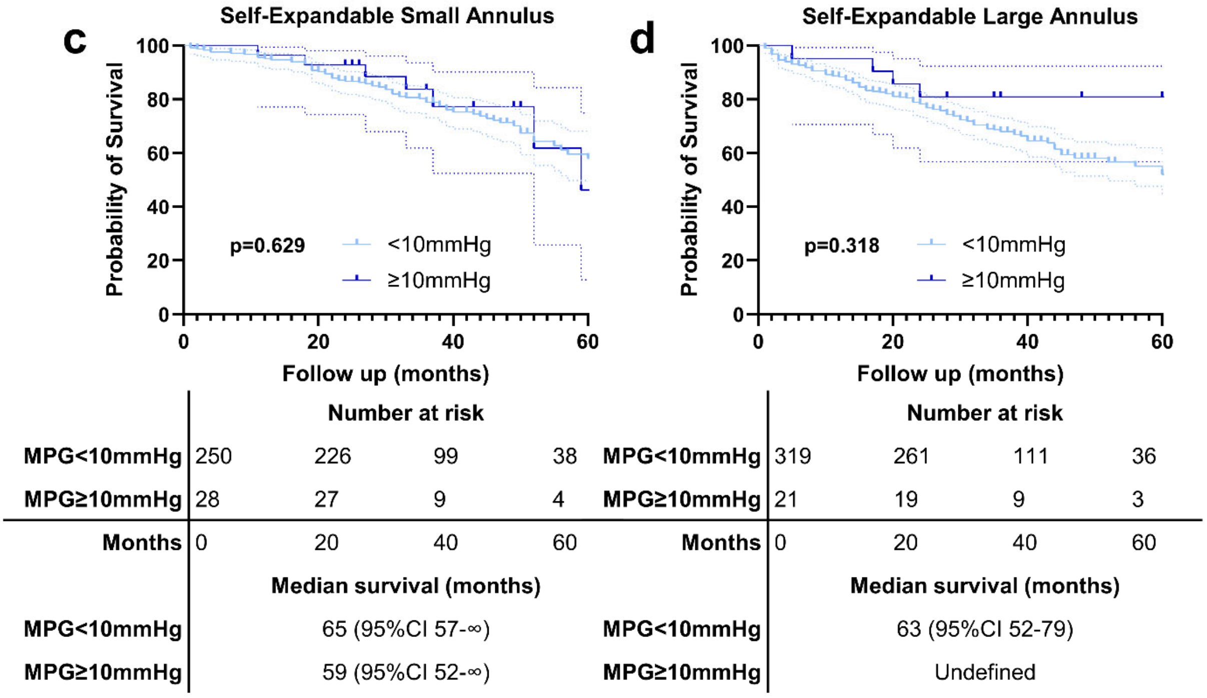

	Number at risk (c)					Number at risk (d)			
MPG<10mmHg	250	226	99	38	MPG<10mmHg	319	261	111	36
MPG≥10mmHg	28	27	9	4	MPG≥10mmHg	21	19	9	3
Months	0	20	40	60	Months	0	20	40	60

	Median survival (months) (c)		Median survival (months) (d)
MPG<10mmHg	65 (95%CI 57-∞)	MPG<10mmHg	63 (95%CI 52-79)
MPG≥10mmHg	59 (95%CI 52-∞)	MPG≥10mmHg	Undefined

Supplemental Figure S4: Comparison of invasively measured DD in the different valve types in patients with small (≤23mm) and large (≥ 24mm) annuli. **(a)** Sapien 3, **(b)** Sapien 3 Ultra, **(c)** Evolut R, **(d)** Evolut Pro, **(e)** Acurate Neo, **(f)** Acurate Neo2

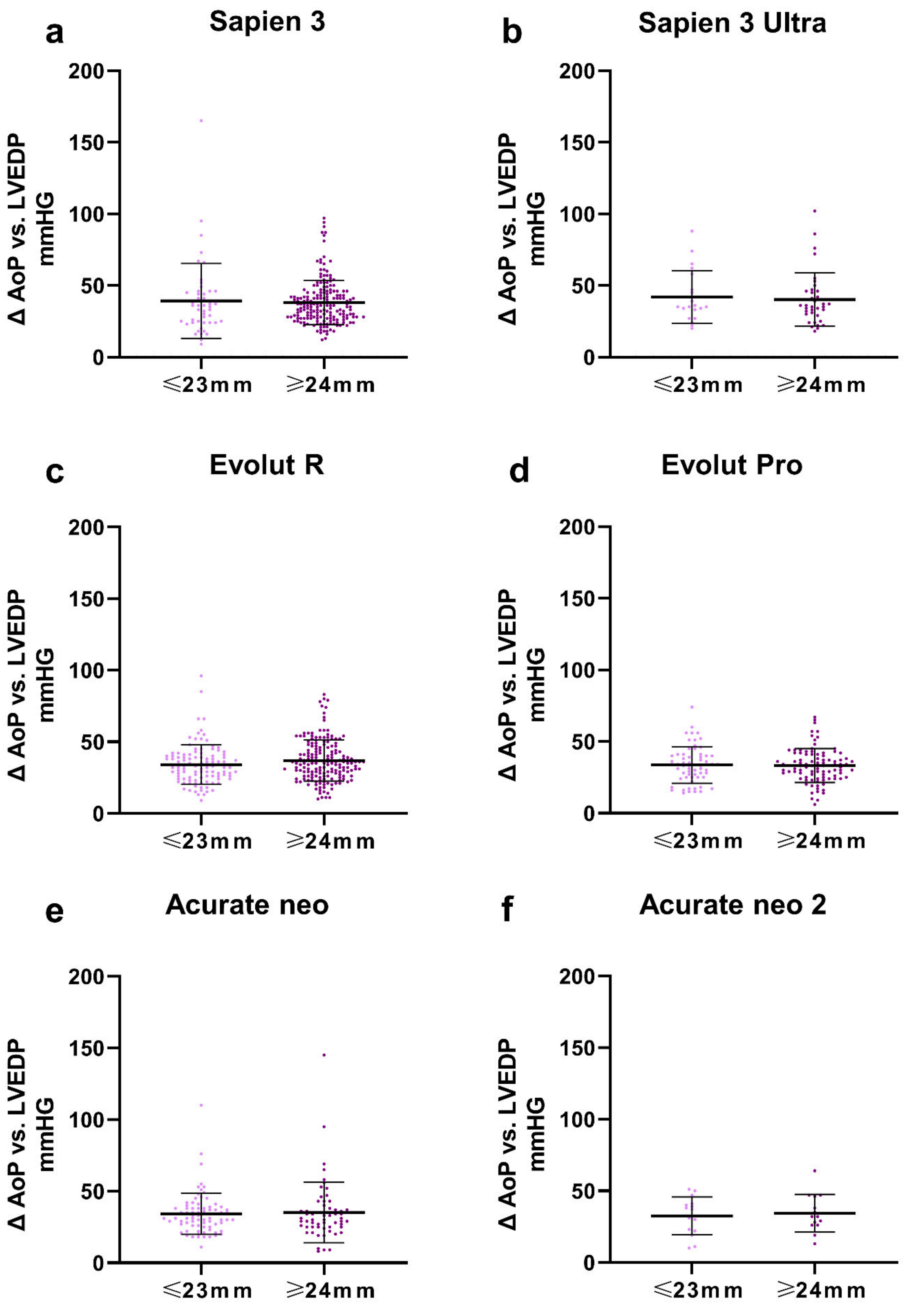

Supplement: Supplementary file 1 [file jcm-14-05875-s001.zip › jcm-3693773-supplementary.pdf]
